# Strength of London Dispersion Forces in Organic Structure Directing Agent—Zeolite Assemblies

**DOI:** 10.3390/molecules29184489

**Published:** 2024-09-21

**Authors:** Karima Ata, Tzonka Mineva, Bruno Alonso

**Affiliations:** ICGM, Université de Montpellier, CNRS, ENSCM, CEDEX 05, 34293 Montpellier, France; karima.ata@umontpellier.fr

**Keywords:** zeolites, organic structure directing agents, host-guest interactions, van der Waals forces, theoretical chemistry

## Abstract

Herein, we study the London dispersion forces between organic structure directing agents (OSDAs)—here tetraalkyl-ammonium or -phosphonium molecules—and silica zeolite frameworks (FWs). We demonstrate that the interaction energy for these dispersion forces is correlated to the number of H atoms in OSDAs, irrespective of the structures of OSDAs or FWs, and of variations in charges and thermal motions. All calculations considered—DFT-D3 and BOMD undertaken by us, and molecular mechanics from an accessible database—led to the same trend. The mean energy of these dispersion forces is ca. −2 kcal.mol^−1^ per H for efficient H-O contacts.

## 1. Introduction

Since their introduction in the 1960s [1,2,3], organic structure directing agents (OSDAs) have become key reactants for zeolite synthesis, allowing researchers to widen the range of accessible framework (FW) types and compositions [4,5,6,7].

Despite the wide use of OSDAs, the understanding of the non-covalent interactions they establish with zeolite frameworks is still a matter of active research. Among these interactions, van der Waals (vdW) forces, and notably London dispersion forces, are recognized to play a significant role in zeolite structure direction [4,8]. A high stabilization of the organic-inorganic assemblies is therefore reachable by these forces through the maximization of contacts between OSDA and zeolite oxide frameworks [4,9]. Meanwhile, this maximization of contacts also favors the formation of stabilizing CH…O weak hydrogen bonds (HBs) [10,11,12,13,14]. Therefore, OSDAs’ characteristics like volume, shape and conformations can indeed promote different pore architectures and zeolite topology [9,15,16,17,18,19,20]. The charges involved in the formation and stabilization of zeolites must also be taken into account, implying a significant contribution of additional polarization and electrostatic interactions [15,21,22,23,24,25]. In some particular cases, other intermolecular interactions come into play like OH…O or NH…O strong HBs [24]. For some systems, OSDA molecules assemble into dimers or larger clusters—through vdW or more specific stacking—that are the final templating species for cages or channels, leading to an even more complex picture [26,27,28]. In addition, dynamic aspects, and notably the motions of OSDAs, should be considered as well [9]. OSDA–zeolite assemblies are therefore the seat of a subtle interplay of intermolecular interactions.

OSDA–zeolite interactions are mostly investigated by theoretical calculations which allow for the quantification of the energy involved, and thus the classification of the organic-inorganic assemblies on the basis of thermodynamic stabilization arguments. Since their development in the 1990s, these calculations are often undertaken to screen and/or identify the best OSDAs for given zeolite framework types in order to favor or discover new zeolite syntheses [17,29,30,31,32,33,34]. For large screenings, the calculation time necessary to minimize the energy of the systems needs to be reduced so as to explore a maximum of OSDA–zeolite systems. In such cases, the calculations use molecular mechanics (MM) and atomic force fields (FF) parameters [30,35,36,37]. Recently, these approaches have been successfully extended to broaden the search for possible OSDAs using machine learning [38], new algorithm procedures [39] and data mining [40,41].

Detailed investigations have also been carried out on specific OSDA–zeolite systems, the list of which will be too long for this manuscript. For instance, calculations using density functional theory with London dispersion correction (DFT-D) allowed researchers to understand how OSDA and F can stabilize a silica FW (ITW) at the expense of another one (TON) that is denser and more stable in its porous form than the former zeolite [11]. Other authors combine inelastic neutron scattering with MM and DFT calculations to discuss the effect of the balance between Coulomb and vdW forces on the motions of OSDAs inside RTH zeolite cavities [23]. The methods for these in-depth studies are based on molecular mechanics or quantum mechanics (DFT) or a combination of both [42,43,44].

Among all these previous studies, there is not a systematic evaluation of the strength of vdW forces between OSDAs and zeolite oxide networks, irrespective of a particular system. Additionally, we have recently taken a new look at previously published data on DFT-D3-optimized OSDA–zeolite assemblies for the study of weak HBs [14], and we noticed that the contribution of the dispersion interaction energy (E^disp^) is almost proportional to the number of hydrogen atoms in the OSDA (n_H_^OSDA^). In the present work, we further investigate these aspects, notably by including additional DFT-D3 calculations and by comparing our calculated vdW interaction energy values to those also obtained here by ab initio dynamics simulations, and to those obtained by MM for other previously studied OSDA–zeolite assemblies [40,41]. As a result, we demonstrate that the minimum interaction energy for dispersion forces between OSDA and silica FW is indeed related to n_H_^OSDA^, and that its value is twice the energy of the dispersion forces between n-alkane molecules. 

## 2. Results

### 2.1. DFT-D3 Calculations

**Scheme 1:**
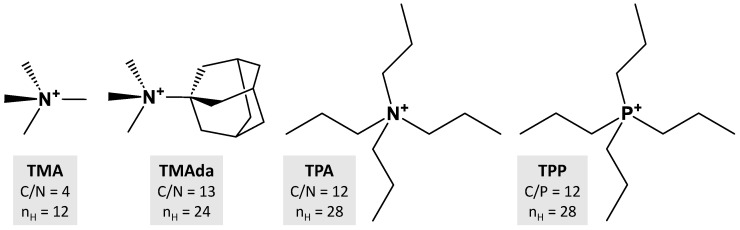
OSDA cations considered for DFT-D3 calculations.

We have undertaken DFT-D3 geometrical optimizations on representative OSDA–zeolite structures and estimated their interaction energies (Table 1). In order to favor OSDA–zeolite contacts and avoid additional OSDA-OSDA interactions, we have excluded systems presenting OSDA clustering. The chosen systems contain a single OSDA molecule per cage, channel or channel crossing. These OSDAs are common tetraalkylammonium or tetraalkylphosphonium cations of different size and structure (Scheme 1) without aromatic rings or organic functions (OH, NH, …) that could lead to additional intermolecular interactions. The zeolite FWs are pure silica with different topologies known to be obtained experimentally through an OSDA templating: AST with tetramethylammonium (TMA@AST), CHA and STT with trimethyladamantylammonium (TMAda@CHA, TMAda@STT) and MFI with tetrapropylammonium (TPA@MFI) and tetrapropylphosphonium (TPP@MFI). The use of a pure silica FW allows us to avoid considering heteroelement siting distributions (e.g., Al^3+^, B^3+^, etc.) that will necessitate much more complex computational considerations, which are unnecessary for our purposes here. Silica FWs contain F atoms to precisely locate the negative charges compensating for the positive charges brought by the OSDAs, and to avoid taking into account SiO^−^ compensating charges whose distribution is more random. In addition, we have optimized one hypothetical structure without charges TMA*@AST starting from TMA@AST, removing F atoms and replacing N by C prior optimization.

The energies presented in Table 1 correspond to the interaction energies between the OSDA and the silica FW containing F, without any OSDA-OSDA or OSDA-F interaction. They are calculated by subtracting the energy of the zeolite containing F and the energy of the OSDA molecules in the crystal cell from the energy of the optimized OSDA–zeolite structure without any further optimization (see Section 3 Methods). The total energy for all intermolecular interactions is labeled as E^tot^. E^disp^ represents the interaction energy due to the London dispersion term that accounts for the majority of the energy involved in vdW forces, notably for interactions between dissimilar or non-polar molecules [45]. The energy of the weak CH…O hydrogen bonds is EHB, and E^other^ represents the energy for the rest of the interactions, mainly electrostatic ones (E^tot^ = E^disp^ + E^HB^ + E^other^).

The resulting interaction energies reveal several trends. In the case of the charged systems, E^tot^ values are three to seven times larger than E^disp^ values because of the known major contribution of electrostatic interactions. This suggests that identifying the OSDA templating ability solely from energy minimization of vdW forces might not be a unique strategy, as also noticed in other works [25]. Besides, weak CH…O HBs lead to a small but non-negligible contribution to the interaction energies, as pointed out earlier [13]: E^HB^ represents 16 to 40% of E^disp^ values. This is also valid for the non-charged hypothetical structures TMA*@AST.

**Figure 1:**
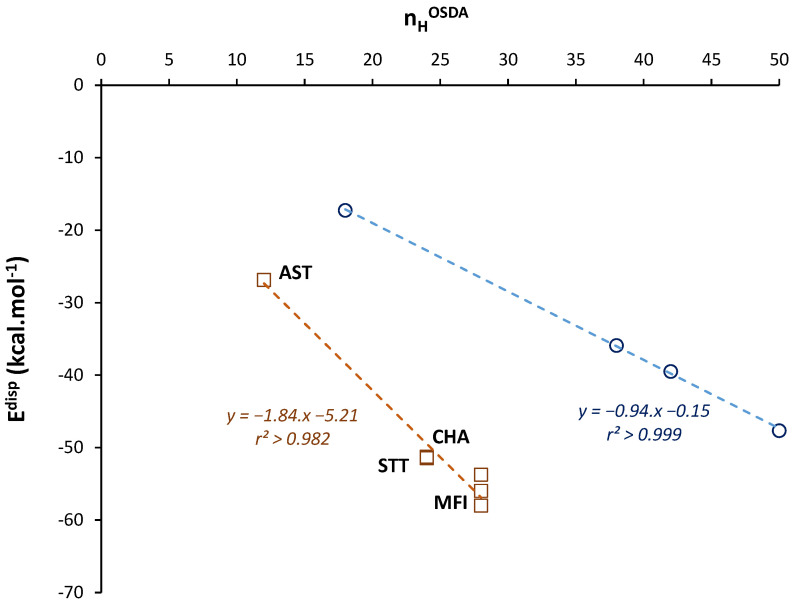
Variations in London dispersion energy E^disp^ as a function of n_H_^OSDA^ in OSDA–zeolite assemblies (squares) and in n-alkanes (circles). E^disp^ values are calculated after DFT-D3 optimizations of the related crystal structures. The dashed lines are obtained by linear regression.

Regarding the OSDA–zeolite dispersion forces, we notice a high similitude for E^disp^ values expressed per moles of H^OSDA^, with values ranging between −1.9 and −2.3 kcal.mol^−1^, including the non-charged structure. The variation of E^disp^ as a function of n_H_^OSDA^ is plotted in Figure 1. Although the number of points is limited, a linear fit of E^disp^ values leads to a high correlation (*r*^2^ > 0.98). The slope is ca. −1.84 kcal.mol^−1^ and corresponds to the increment in dispersion energy per each H atom of the OSDA.

### 2.2. Comparisons with n-Alkanes

To validate this estimation, we also present in Figure 1 the E_disp_ variations as a function of the number of H atoms in n-alkanes of different lengths. In this case, the slope of the linear plot (*r*^2^ > 0.999) is −0.94 kcal.mol^−1^. This value is close to the increment in vdW energy previously estimated or measured for n-alkanes (ca. −0.82 kcal.mol^−1^ per H) [45]. The slightly higher absolute value obtained here (ca. +14%) is explained by the known energy overestimation of the D3 approach [46]. From this global agreement for n-alkanes, it appears therefore that the variations in E^disp^ for OSDA–zeolite assemblies are giving a realistic picture of the interaction energy through van der Waals forces. 

We notice a ca. 50% smaller absolute value for dispersion energies between n-alkanes compared to that between OSDA and the silica FW. We can try to rationalize this difference by considering as a first approximation the London energy term for two atoms in the cases of H-H and H-O intermolecular contacts. The London’s expression is then

E^disp^ = −3/2(I_1_.I_2_/(I_1_ + I_2_)).(α_01_.α_01_/(4πε_0_)^2^).d_12_^−6^ (1)

where I_i_, α_0i_ and d_12_ are the first ionization potentials, the electronic polarizabilities and the intermolecular distance [47]. First ionization potentials are very close for H and O (difference < 0.2%) [48], and the ratio between dispersion energies can be written as

E^disp^(H-H)/E^disp^(H-O) ≈ (α_0_{H}/α_0_{O}).(d_HH_/d_HO_)^−6^ (2)

From DFT-D3 calculations, the intermolecular H-H and H-O contact distances (distances between first neighbors) can take similar values in n-alkanes crystals and in OSDA–zeolite assemblies (Figures S1 and S2). The presence of short H-O contacts in OSDA–zeolite assemblies is explained by the combination of several attractive intermolecular interactions: for instance, Coulomb electrostatic forces and weak CH…O HBs in addition to dispersion forces.

If we make the crude hypothesis of *d*_HH_/*d*_HO_ ≈ 1, the energy ratio would then mostly depend on the ratio between electron polarizabilities. Considering that the electron polarizabilities reported for C-H bonds and Si-O-Si bonds (α_0_ = 0.65 and 1.39 (4πε_0_)10^−30^ m^3^, resp.) [45] correspond here to α_0_{H} and α_0_{O}, we end up with E^disp^(HH)/E^disp^(HO) ≈ 0.5. This is obviously a rough estimation that shows the relevance of electron polarizabilities to explain the remarkable contrast in dispersion energies in the case of n-alkane and OSDA–zeolite crystals. It also highlights the great attraction existing between alkyl groups and siloxane bonds forming OSDA–zeolite assemblies.

Since experimental measurements of E^disp^ for OSDA–zeolite interaction are not physically accessible, calorimetric studies of n-alkane adsorption in zeolites can be used indirectly to judge the relevance of the calculations performed. A prior study compared the heats of adsorption of methane, ethane and n-propane in zeolites of contrasted pore geometry and pore diameter [49]. For the zeolite FW presenting the smallest average pore diameter (FER)—that would allow a high number of H-O contacts—the incremental increase in isosteric heat of adsorption per H is 1.53 kcal.mol^−1^. This value is significantly higher than the absolute value of experimental vdW energy between n-alkanes (vide supra), confirming the higher attraction of n-alkanes towards zeolite FWs. It is still smaller than the absolute values for the E^disp^ increments (ca. 1.84 kcal.mol^−1^) calculated here for dispersion interactions between OSDA and zeolite FWs. Nevertheless, the ca. 20% deviation could be explained by less efficient H-O contacts in the case of n-alkane adsorption compared to OSDA templating, and by a slight overestimation of E^disp^ computed with D3 as mentioned above.

### 2.3. Effects of Framework, Composition, Charges and Motions

It is also interesting to note that the E^disp^ variations are obtained for OSDAs of different structures (although belonging to the family of tetraalkyl-ammonium/-phosphonium cations) and for zeolites of completely different framework types and pore geometry (Table 2). An example of that is the almost identical E^disp^ value calculated for TMAda molecules in CHA and STT zeolite hosts. Also interesting are the calculations for TPA and TPP cations in MFI zeolites (*Pnma* space group), which give similar E^disp^ values. The slightly smaller value for TPP (ca. 7% deviation) can be understood through the effect of longer P-C bonds (1.82–1.84 Å) compared to N-C in TPA (1.53–1.55 Å) giving rise to slightly shorter H-O contacts (smallest H-O distances are 2.43 and 2.47 Å for TPP@MFI and TPA@MFI, resp.) and increasing HB formation. 

Additionally, the dispersion energy remains the same in TMA@AST and in the hypothetical TMA*@AST structure when the formal charges of the OSDA–zeolite assembly are removed. In this latter case, the calculated interaction energy E^other^ is almost zero. These results are expected. They confirm that the calculated E^disp^ values are fully uncoupled from the Coulomb electrostatic interactions.

An additional important aspect we want to investigate is the effect of thermal motions. For this purpose, we used BOMD dynamics simulations for three selected systems: TMA inside AST cages, TMAda inside STT cavity and TPA at the crossing of MFI channels. The BOMD dynamics are performed at a temperature of 300 K (see Methods). After the thermal equilibration, four structures (snapshots) were selected at every ps along the trajectories from the 2nd and 5th ps of the simulation time for each OSDA–zeolite FW system. The interaction energies in the selected snapshots are calculated from the single-point SCF calculations (without further structural relaxation) with Crystal23 code using Equation (5) in the Methods section below. For a given system, the resulting values of E^disp^ are all almost identical (maximum deviation < 2%) to those of the optimized structure (Table 1). This demonstrates that there is no major effect of thermal motions on the overall strength of London dispersion forces (the crystal cell parameters being always set to those of the DFT optimized structures, Table 3 in Methods).

Consequently, all these results based on DFT-D3 and BOMD calculations suggest the existence of a universal trend in the dispersion energy between OSDAs and zeolite structures, independent of chemical peculiarities and molecular dynamics. The verification of this hypothesis necessitates working with a significantly higher number of OSDA–zeolite assemblies, something not reachable by DFT geometrical optimizations. We therefore turned to the use of structures obtained by molecular mechanics calculations.

### 2.4. Comparison with MM Calculations

**Figure 2:**
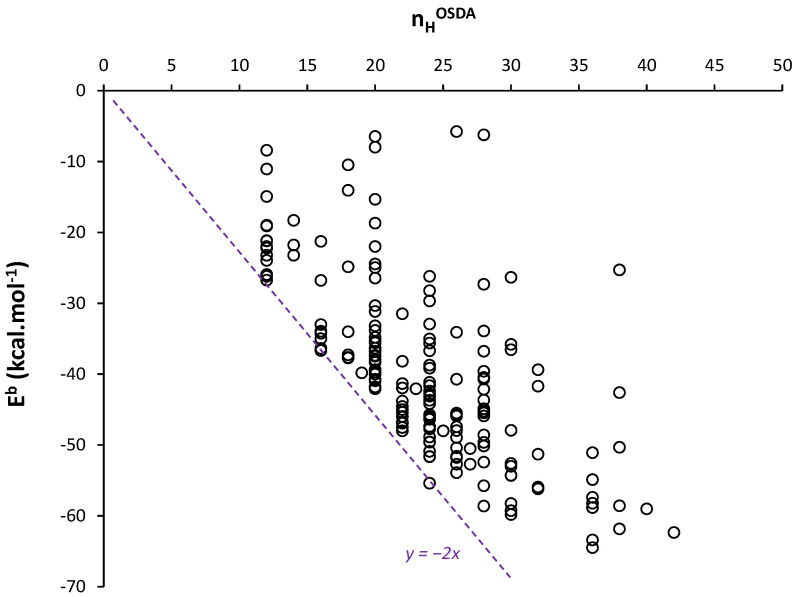
Variations in binding energy E^b^ as a function of n_H_^OSDA^ in OSDA–zeolite assemblies. E^b^ values are obtained from the OSDB and MM calculations [41,50]. The dashed line is a guide for eyes.

The OSDB database has been generated from MM calculations [41,50]. This database presents interaction energies, expressed as binding energies E^b^, that estimate vdW interaction energies between OSDA and silica zeolite FW, for thousands of possible OSDA-FW pairs. Figure 2 presents the binding energy E^b^ per OSDA as a function of n_H_^OSDA^ for a non-exhaustive and random set of 192 zeolite-OSDA structures extracted from the OSDB database and made of quaternary ammonium OSDAs considered as neutral, without any other heteroatom (e.g., O). The observed dispersion in E^b^ values might be explained by the different templating ability of the OSDA with respect to the zeolite FWs. More importantly, we observe that the minimum values of E^b^ do not exceed a limit that depends on n_H_^OSDA^ (visualized by the dashed line in Figure 2). This limit corresponds here to a maximization of vdW contacts between the surface of the OSDA—formed by the shell of H atoms—and the silica FW.

**Figure 3:**
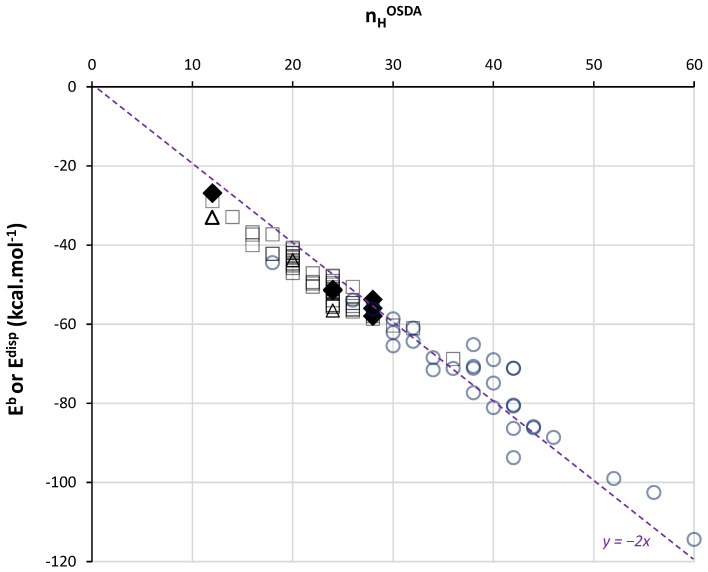
Minimum binding energy *E*^b^ obtained from OSDB [50] (empty squares for quaternary ammoniums, empty circles for diquaternary ammoniums and empty triangles for tetraalkylphosphoniums) and dispersion interaction energy *E*^disp^ obtained by DFT-D3 calculations (filled lozenges) as a function of the number of H in each OSDA. The dashed line is a guide for the eyes.

Further, we consider the minimum values of E^b^ per OSDA whatever the silica FW type. This allows us to deal with energies corresponding to a maximum of vdW contacts. Figure 3 presents minimum E^b^ values extracted from OSDB for 90 quaternary and diquaternary ammonium and phosphonium OSDAs as a function of n_H_^OSDA^ (empty marks). The correlation of a linear regression is very high (*r*^2^ > 0.95) (Figure S3A). It is also noteworthy that this correlation translates into a correlation between minimum E^b^ values and the estimated molecular volume of the OSDA (Figure S3B), in line with other recent work showing the relationship between vdW interaction energy and OSDA sizes [51]. Indeed, the considered OSDAs present a C-N or C-P skeleton on which H atoms are attached, forming the external shell that defines the OSDA’s volume.

The E^disp^ values obtained by DFT-D3 in this work (Figure 1) are compared to these minimum E^b^ values in the same Figure 3. We observe that both sets of DFT-D3 and MM values match perfectly. It has to be noted that the methodology followed for MM calculations was selected by its ability to reproduce DFT-D3 energy calculations (using in this case a plane-wave approach) [52]. The important point here is the confirmation of a universal trend in the vdW interaction energy as a function of n_H_^OSDA^. From Figure 3, we remark that small OSDAs (n_H_^OSDA^ < 20) lead to stronger dispersion forces per H^OSDA^ (ca. −2.3 kcal.mol^−1^). In this case, the OSDA molecules are surrounded by siloxane bonds, and most of the H atoms, if not all, are in contact with the silica FW. For larger OSDAs, and larger cages or channels, the contact between H atoms and silica is probably less complete, leading to a slight decrease in the strength of dispersion forces per H. 

The estimated range for dispersion OSDA–zeolite energies per H^OSDA^ (ca. −2 kcal.mol^−1^) agrees relatively well with some other recent works based on tetraalkylammonium OSDAs [53,54]. However, a general comparison with a wider range of previously calculated datasets is more erratic. This can be attributed to differences in force field parameters, or DFT methods, as well as on the templating effect of the OSDAs.

## 3. Methods

### 3.1. Geometry Optimizations

The experimental OSDA–zeolite crystallographic structures were optimized using a periodic density-functional theory (DFT) approach and all-electron atomic centered wave functions at a theoretical temperature of 0 K with the CRYSTAL17 program [55]. Only the atomic positions were optimized to avoid the overexpansion of the cell parameters. Table 3 summarizes the initial structural parameters considered for the OSDA–zeolite assemblies [56,57,58,59,60].

**Table 3:** Characteristic properties of initial OSDA–zeolite crystallographic structures.

System	Space Group	*a* (Å)	*b* (Å)	*c* (Å)	*α* (°)	*β* (°)	*γ* (°)	*V* (Å^3^)	*F* Location	Ref.
TMA@AST	*I*4/*m* ^a^	9.0680	9.0680	13.4379	90	90	90	1104.98	[46]	[56]
TMAda@CHA	*R*-32/*m* ^a^	13.4902	13.4902	14.7578	90	90	120	2325.89	[4662]	[57]
TMAda@STT	*P*12_1_/*n*1	12.9594	21.7919	13.5980	90	101.9	90	3758.30	[5443]	[58]
TPA@MFI	*Pn*2_1_*a* ^b^	19.7271	19.8624	13.3155	90	90	90	5217.38	[415262]	[59]
TPA@MFI	*Pnma*	20.0026	19.9934	13.3923	90	90	90	5355.85	[415262]	[60]
TPP@MFI	*Pnma*	20.0706	19.9733	13.4017	90	90	90	5372.42	[415262]	[60] ^c^

^a^ To individualize C and H positions, which are otherwise multiple due to symmetry elements, the space group considered for calculations was *P*1. ^b^ For TPA@MFI with a *Pn*2_1_*a* space group, cell parameters are obtained from a preliminary optimization [59]. ^c^ The TPA@MFI structure of reference [60] was considered as initial input with cell parameters re-adjusted from a TPP@MFI experimental XRD pattern.

In order to decouple the Coulomb electrostatic interactions from the vdW and weak HBs interactions, we constructed a hypothetical non-charged TMA*-AST model structure. This structure was built using the same initial structural parameters of TMA@AST, removing F from the [46] cages and replacing N in TMA by a C atom. The atomic positions of the resulting structure were then re-optimized.

The n-alkane crystallographic structures considered in this study, available at the Cambridge Structural Database, are: n-octane [61], n-eicosane [62], n-octadecane [63] and n-tetracosane [64]. The atomic positions of these systems were optimized under the same computational conditions as mentioned below.

Based on the benchmark results of preliminary calculations on the AST@TMA structure [14] varying basis sets, approximations for dispersion correction and using gradient-corrected (PBE) and hybrid (B3LYP) exchange-correlation (XC) functionals, the following computational setup was chosen to study OSDA–zeolite systems: the periodic DFT-D3 approach with the empirical London dispersion (D3) term [65] and the Becke-Johnson damping function incorporated [66] according to the scheme implemented in the CRYSTAL17/23 codes [55,67]. For the optimization, all-electron atomic-centered wave functions of triple-ζ quality (TZVP) [68] were used. Following the work of Zicovich-Wilson et al. [11], the use of TZVP yielded also very good agreement between Si-O distances and O-Si-O angles in zeolites determined theoretically and experimentally. The XC functional applied was the generalized gradient-corrected PBE approximation [69].

### 3.2. Dispersion Corrections

The calculations incorporate a London dispersion term through the D3 approach based on a parametrization of dispersion coefficients with improvements regarding the description of atom-pairwise dispersion coefficients and cutoff radii [65]. This approach is currently considered one of the most commonly used approaches to add the dispersion term to the DFT energy and energy gradients [70]. It is adapted for alkyl groups [46] and neutral or ionic complexes not containing metal cations [71]. Although adsorption energies of small molecules inside zeolites might be overestimated by D3 [72,73,74,75], the PBE-D3 combination is found to be a good compromise in terms of DFT computational cost and accuracy [52].

We have verified that empirical and semi-classical corrections D2 [76] do not affect the interaction energies much compared to D3. This is shown in Table 4 for the TMA@AST system, where the calculated interaction energies of the optimized structures (PBE-TZVP and atoms only) using both D2 and D3 show minimal differences. In fact, E^disp^ shows only a 4% decrease in absolute values when using D2 compared to D3.

### 3.3. Interactions Energies

The interaction energies are calculated using the optimized structures, taking into account the energy of the OSDA–zeolite-F system ($E_{zeolite-OSDA-F}$), the silica framework with F ($E_{zeolite-F}$) and the OSDAs in the crystal cell ($E_{OSDAs}$). The energies of each fragment were obtained from a single-point self-consistent field (SCF) calculation using the revised wave functions of triple-ζ quality (TZVP-REV2) that were demonstrated to be free from the basis set superposition error (BSSE) [77].

The total interaction energy per OSDA (E^tot^/OSDA) and per Si (E^tot^/Si) is calculated as follows:

(3) $$\frac{E^{tot}}{OSDA}=\frac{1}{n}\left(E_{zeolite-OSDA-F}^{tot}-E_{zeolite-F}^{tot}-E_{OSDAs}^{tot}\right)$$

(4) $$\frac{E^{tot}}{Si}=\frac{1}{m}\left(E_{zeolite-OSDA-F}^{tot}-E_{zeolite-F}^{tot}-E_{OSDAs}^{tot}\right)$$

where *n* and *m* are the number of OSDAs and Si atoms in the crystal unit cell, respectively.

Similarly, the dispersion interaction energy between OSDA and zeolite is calculated using

(5) $$\frac{E^{disp}}{OSDA}=\frac{1}{n}\left(E_{zeolite-OSDA-F}^{disp}-E_{zolite-F}^{disp}-E_{OSDAs}^{disp}\right)$$

(6) $$\frac{E^{disp}}{Si}=\frac{1}{m}\left(E_{zeolite-OSDA-F}^{disp}-E_{zolite-F}^{disp}-E_{OSDAs}^{disp}\right)$$

The hydrogen bond energy for each system is calculated from the local electron kinetic density *G* and the Laplacian of the electron density ∇^2^*ρ* at the bonding critical point (3, −1) defining the HBs. These values are obtained through the charge density Topological Analysis using the TOPOND package [78]. The total hydrogen bond energy formed by H atoms of one OSDA in the crystal unit cell $E^{HB}/OSDA$ is the sum of each individual HB energy ($E^{HB}$), following

(7) $$E^{HB}=\frac{h^{2}}{4m}\nabla^{2}\rho \left(r_{CP}\right)-2G\left(r_{CP}\right)$$

(8) $$\frac{E^{HB}}{OSDA}=\sum_{rcp}E^{HB}$$

where r_CP_ corresponds to the position of the critical point and *m* to the electron mass.

### 3.4. Dynamics Simulation

Dynamic simulations, starting with each optimized structure as described above, were carried out using Born-Oppenheimer molecular dynamics (BOMD) with the Quantum Espresso (QE) code [79,80]. The same cell parameters presented in Table 3 were used. The Bravais lattice was set to type 0, which corresponds to space group *P*1. The PBE exchange-correlation functional with the D3 London dispersion correction was used. All atoms were described by ultrasoft pseudopotentials [81]. The energy cutoffs were 60 and 720 Ry for the wave functions and charge density energy, respectively. The energy convergence threshold was set to 10^−7^ a.u. The temperature was controlled at 300 K with a Berendsen thermostat that ensures a rapid thermalization to the target temperature. The time constant was set to 4 times the integration step (Δ*t*) for all the calculations. The default integration step Δ*t* = 0.96 fs in the Quantum Espresso software was used with the velocity-Verlet algorithm [82,83] for integrating the equations of motion at each iteration. Each OSDA–zeolite system was simulated for 5 ps in order to generate various OSDA–zeolite configurations.

## 4. Conclusions

Herein we demonstrate that the interaction energy for London dispersion forces between OSDA and silica FW decreases linearly with nHOSDA (number of H atoms in OSDA) for non-clustered and fully templating tetraalkyl-ammonium or -phosphonium OSDAs, irrespective of the specific structures of the OSDAs or FWs. This relationship is also independent of the formal charges borne by the OSDA as shown for a hypothetical non-charged assembly. Further, the relationship is unchanged when considering the thermal motions of the host-guest assemblies.

From DFT-D3, BOMD and MM calculations, we estimated the energy of these London dispersion forces to be ca. −2 kcal.mol^−1^ per H for efficient H-O contacts. When such values are reached, the effect of van der Waals forces is maximal and the OSDA is fully acting as a template. This might be used as a complementary criterion when analyzing OSDA–zeolite assemblies.

Furthermore, we show that the estimated energy for dispersion forces between OSDA and silica is two times stronger than that between n-alkanes (in absolute values). This is attributed to the differences in electron polarizabilities and highlights the high attraction existing between alkyl groups and siloxane bonds in OSDA–zeolite host-guest assemblies.

The strength found here for the dispersion forces between OSDAs and silica FWs confirms that vdW forces are undoubtedly a major intermolecular interaction to consider for understanding OSDA–zeolite assemblies and other host–guest zeolite-based structures. Among the non-covalent interactions that govern the OSDA–zeolite assemblies, we show, however, that other intermolecular interactions can have greater energy than dispersion forces. Obviously, this does not imply that vdW forces, nor weak CH…O H-bonds, do not play an important role in structure direction and phase selectivity, but care has to be taken when discriminating OSDA–zeolite assemblies using simplified approaches that might neglect some important electrostatic interactions.

The final picture that emerges is the presence of strongly interacting OSDAs and silica zeolite FWs thanks to a combination of several attractive intermolecular interactions that shorten H-O contacts, leading to the high absolute values estimated here for the interaction energy of London dispersion forces.

## Figures and Tables

**Table 1:** Calculated interaction energies of OSDA–zeolite intermolecular interactions (in kcal.mol^−1^).

	E^tot^/Si	E^disp^/Si	E^tot^/OSDA	E^disp^/OSDA	E^HB^/OSDA	E^other^/OSDA	E^tot^/H	E^disp^/H
TMA@AST	−17.7	−2.7	−177.2	−26.9	−6.6	−143.8	−14.77	−2.24
TMAda@CHA	−13.1	−4.3	−156.9	−51.2	−20.0	−85.6	−6.54	−2.13
TMAda@STT	−11.6	−3.2	−185.1	−51.5	−10.9	−122.7	−7.71	−2.14
TPA@MFI (*Pn*2_1_*a*)	−9.0	−2.3	−217.0	−56.0	−8.8	−152.2	−7.75	−2.00
TPA@MFI (*Pnma*)	−8.9	−2.2	−214.2	−53.7	−11.2	−149.3	−7.65	−1.92
TPP@MFI (*Pnma*)	−8.9	−2.4	−213.9	−58.0	−12.8	−143.1	−7.64	−2.07
TMA*@AST ^a^	−3.1	−2.7	−31.1	−26.9	−7.0	2.8	−2.59	−2.24

^a^ Hypothetical structure with uncharged atoms (see Section 3 Methods).

**Table 2:** Calculated interaction energies of dispersion forces between OSDA and zeolite FW for DFT optimized structures and snapshots selected along the trajectories of the BOMD dynamics simulations (in kcal.mol^−1^).

Zeolite FW Type	AST	STT	MFI
OSDA	TMA	TMAda	TPP
Initial DFT-D3 optimization			
Space group	*P*1	*P*12_1_/*n*1	*Pn*2_1_*a*
E^disp^/OSDA (E^disp^/H)	−26.9 (−2.24)	−51.5 (−2.14)	−56.0 (−2.00)
BOMD simulation T = 300 K			
Space group	*P*1	*P*1	*P*1
E^disp^/OSDA (E^disp^/H) at *t* = 2 ps	−27.2 (−2.27)	−51.7 (−2.15)	−56.8 (−2.03)
E^disp^/OSDA (E^disp^/H) at *t* = 3 ps	−27.0 (−2.25)	−51.6 (−2.15)	−56.4 (−2.01)
E^disp^/OSDA (E^disp^/H) at *t* = 4 ps	−27.2 (−2.27)	−51.7 (−2.16)	−56.6 (−2.02)
E^disp^/OSDA (E^disp^/H) at *t* = 5 ps	−27.1 (−2.26)	−51.6 (−2.15)	−56.2 (−2.01)

**Table 4:** Interaction energies for the TMA-AST system optimized using D2 and D3 dispersion.

Interaction Energies (in kcal.mol^−1^)				
	E^tot^/OSDA	E^disp^/OSDA	E^tot^/H	E^disp^/H
TMA@AST (D3)	−177.2	−26.9	−14.77	−2.24
TMA@AST (D2)	−175.2	−25.8	−14.60	−2.15

## Data Availability

Data are contained within the article and Supplementary Materials.

## Supplementary Materials

The following supporting information can be downloaded at: https://www.mdpi.com/article/10.3390/molecules29184489/s1, Figure S1: Examples of H-H distance distributions in DFT-D3 optimized structures of n-octane and n-tetradecane; Figure S2: Examples of H-O distance distributions in DFT-D3 optimized structures of OSDA–zeolites assemblies; Figure S3: Minimum binding energy E^b^ between OSDA and zeolite framework obtained for quaternary ammoniums, diquaternary ammoniums and tetraalkylphosphoniums from the OSDB database [14,41].
