# Supplementary material for: Strength of London Dispersion Forces in Organic Structure Directing Agent—Zeolite Assemblies
Source: Molecules. 2024 Sep 21;29(18):4489. doi: 10.3390/molecules29184489 (PMC11434474; doi:10.3390/molecules29184489)
Supplement: Supplementary file 1 [file molecules-29-04489-s001.zip › molecules-3218310-supplementary.pdf]

- Supplementary data -

Strength of London dispersion forces in organic structure directing agent – zeolite assemblies

Karima Ata, Tzonka Mineva*, Bruno Alonso*

ICGM, Université de Montpellier, CNRS, ENSCM, 34293 Montpellier cedex 5, France.

* Corresponding authors: tzonka.mineva@enscm.fr and bruno.alonso@enscm.fr

Contents:

1. **Comparison of H-H and H-O contacts in n-alkanes and OSDA-zeolite structures**

   Fig. S1 p. 2

   Fig. S2 p. 3

2. **Variations in minimum binding energies between OSDA and zeolite frameworks**

   Fig. S3 p. 4

3. **References** p. 5

1. Comparison of H-H and H-O contacts in n-alkanes and OSDA-zeolite structures

The shortest H-H contacts in n-alkanes structures are intramolecular. The H-H intermolecular contacts appear at larger distances. In the case of the DFT-D3 optimized crystal structures of n-alkanes, H-H intermolecular distances are above 2.3 Å for CH_3 groups and above 2.4 Å for CH_2 groups in the middle of the alkyl chains (see **Fig. S1** below for examples of H-H distance distributions in n-octane and n-tetradecane).

For OSDA-zeolites crystal structures, the shortest H-O contacts appear at values above 2.4 Å (see **Fig. S2** below for examples of H-O distance distributions in TMA@AST, TMAda@STT, TPP@MFI, and also reference [1] for more complete distributions).

See Methods section in the main text for details about calculations.

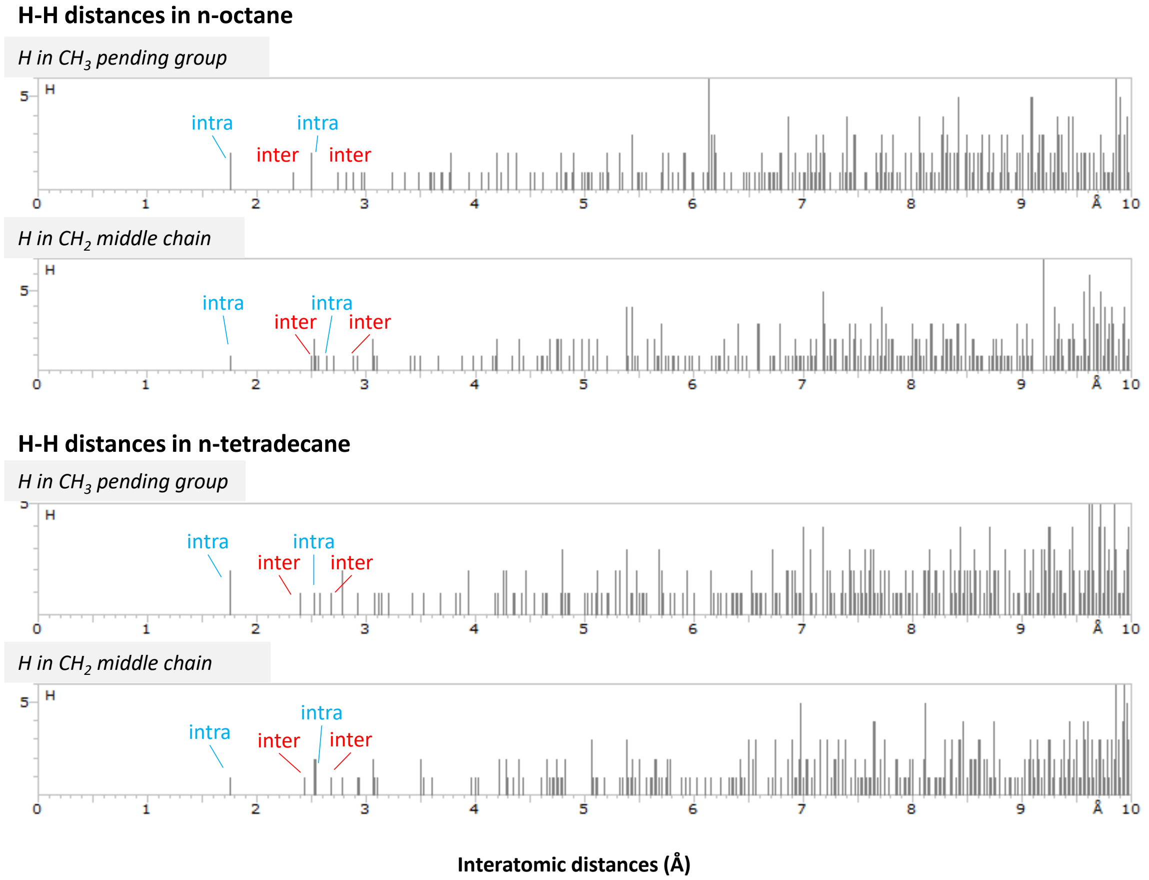

Fig. S1 Examples of H-H distance distributions in DFT-D3 optimized structures of n-octane and n-tetradecane. The intra- or inter-molecular nature of the H-H contacts is given for the smaller distances. Red colors correspond to intermolecular contacts.

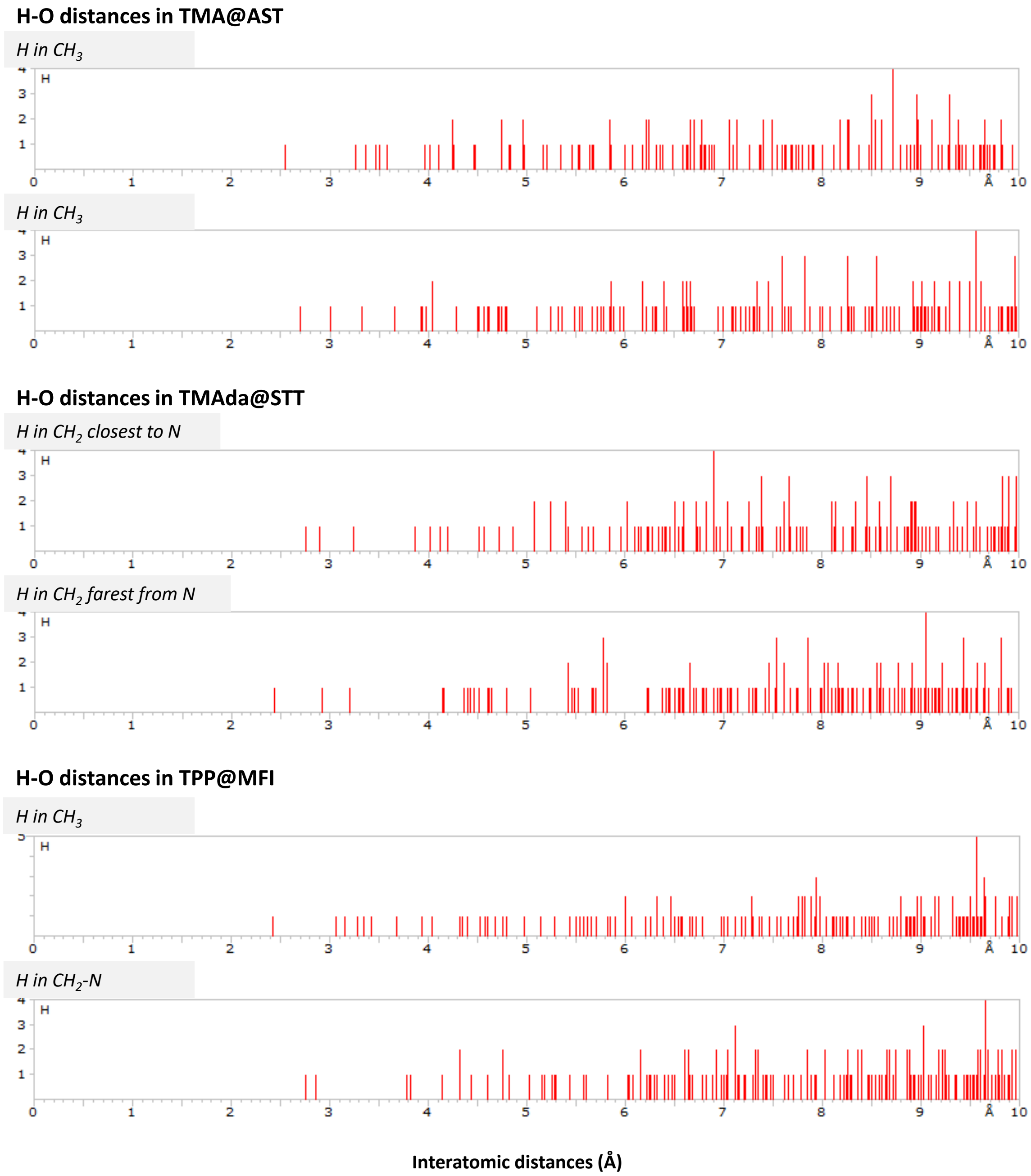

Fig. S2 Examples of H-O distance distributions in DFT-D3 optimized structures of OSDA-zeolites assemblies: TMA@AST, TMAda@STT and TPP@MFI.

2. Variations in minimum binding energies between OSDA and zeolite frameworks

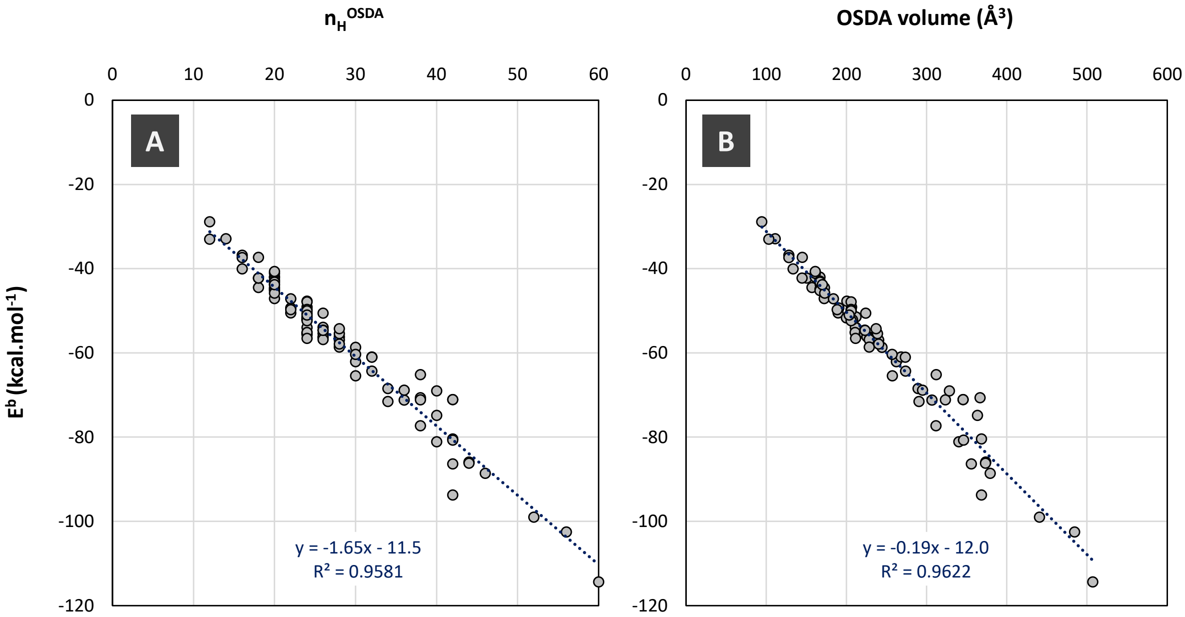

Fig. S3 Minimum binding energy E^b between OSDA and zeolite framework obtained for quaternary ammoniums, diquaternary ammoniums and tetraalkylphosphoniums from the OSDB database [2] as a function of (A) the number of H in OSDA n_H^{OSDA}, and (B) the volume of the OSDA. The equation and the coefficient of determination R^2 correspond to linear regressions of the data (dashed lines).

3. References

[1] S. Al-Nahari, K. Ata, T. Mineva, B. Alonso, Ubiquitous presence of intermolecular CH…O hydrogen bonds in as-synthesized host-guest zeolite materials, Chem. Select 6 (2021) 9728-9734. https://doi.org/10.1002/slct.202102771

[2] D. Schwalbe-Koda, S. Kwon, C. Paris, E. Bello-Jurado, Z. Jensen, E. Olivetti, T. Willhammar, A. Corma, Y. Román-Leshkov, M. Moliner, A priori control of zeolite phase competition and intergrowth with high-throughput simulations, Science 374 (2021) 308-315. https://doi.org/10.1126/science.abh3350
